# Obstructive Sleep Apnea: An Unusual Cause of Hemorrhagic Stroke

**DOI:** 10.7759/cureus.1718

**Published:** 2017-09-27

**Authors:** Nilesh H Pawar, Jennifer A O'Riordan, Preeti Malik, Farhad F Vasanwala

**Affiliations:** 1 Department of General Medicine, Sengkang General Hospital, Sengkang Health, Singhealth, Singapore; 2 Rehabilitation, Changi General Hospital; 3 PUBLIC Health, Icahn School of Medicine at Mount Sinai, New York, N.Y., USA

**Keywords:** stroke, hemorrhagic stroke, obstructive sleep apnea, basal ganglia bleed

## Abstract

Stroke is one of the most common causes of mortality and morbidity worldwide. Hemorrhagic stroke comprises 10-20% of strokes. Here, we present a case report of hemorrhagic stroke that may have been secondary to untreated Obstructive Sleep Apnea (OSA) in a young man with no other cardiovascular risk factors or features of metabolic syndrome. A 32-year-old man was admitted for hemorrhagic stroke. An initial thorough workup for the etiology of stroke was inconclusive. Eventually, a polysomnography was done, which demonstrated OSA suggesting that untreated OSA may have contributed to his stroke. OSA may cause hemorrhagic stroke by nocturnal blood pressure surge. So, all physicians should consider doing polysomnography for unexplained hemorrhagic stroke or in patients at risk. Diagnosing and treating OSA would be critical in preventing hemorrhagic stroke and its recurrences.

## Introduction

Stroke is the second common cause of mortality and morbidity worldwide; hemorrhagic stroke contributes to 10-20% of all strokes [1]. Hypertension is a well-established risk factor for hemorrhagic stroke [2]. In some patients, the etiology remains unclear and bizarre. This is a report on one such patient, who did not have any apparent risk factors. The association between ischemic stroke and obstructive sleep apnea (OSA) has been known since 1999 [3] and several studies have examined the relationship between OSA and ischemic stroke using the Apnea-Hypopnea Index (AHI) as the measurement tool [4]. The AHI measures the severity of sleep-disordered breathing (SDB) by the number of apnea and hypopneas per hour of sleep [4]. Here we describe a case of hemorrhagic stroke possibly caused because of untreated OSA in the absence of any risk factors.

## Case presentation

A 32-year-old Chinese man presented to the emergency department at 3 AM, with weakness and inability to mobilize resulting in a fall. His symptoms began as a sudden onset of left-sided headache, which started four hours earlier during sexual intercourse. Following this, he developed right-sided weakness, facial droop and two episodes of vomiting. The weakness was profound four hours later, resulting in an inability to walk, which subsequently led to a fall.

On examination, body mass index was 23.3 kg/m^2^ and blood pressure (BP) was 135/93 mm Hg. He was drowsy but rousable, Glasgow Coma Scale score was 11/15 [E4V1M6]. Pupils were symmetric and equally reactive to light. The extra-ocular range of movement was full, with no nystagmus. Motor power on the left was 5/5 in both upper and lower extremities. Best motor power in the right upper extremity was 2/5, while in the lower extremity it was 1/5. Deep tendon reflexes were within normal limits. Interestingly, his father was advised to undergo a sleep study in view of presumptive OSA by his primary care physician, however, he declined to proceed.

**Figure 1:**
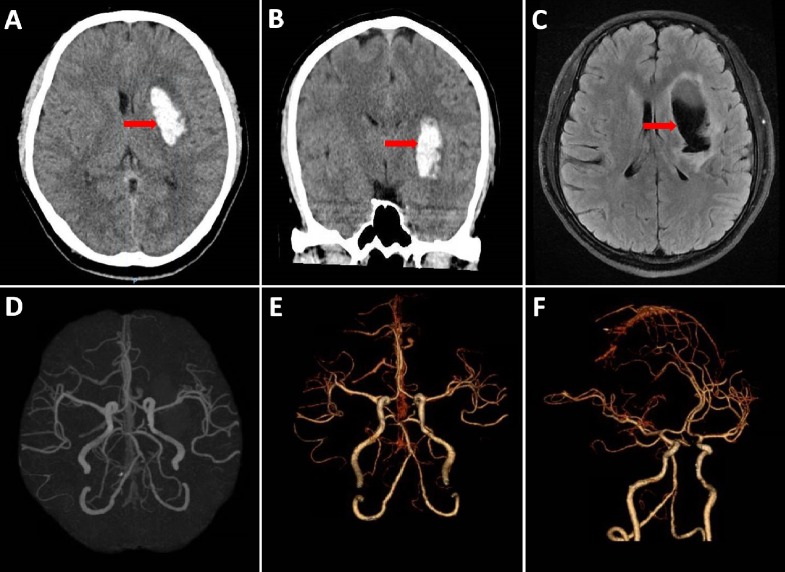
Initial Computed Tomography of Head Axial view (A), Coronal view (B) and Magnetic Resonance Imaging of Brain T2 Axial Flair (C) showing acute intraparenchymal hemorrhage (red arrow) in the left basal ganglia region. Computed Tomography Angiography of Circle of Willis (D-F) showing normal study (no aneurysm or arteriovenous malformation).

Initial computed tomography (CT) of the head and later magnetic resonance imaging (MRI) of the brain showed an acute intraparenchymal hemorrhage in the left basal ganglia with mild perilesional edema, without any midline shift (Figure 1 [A-C]). Blood investigations were unremarkable. Cardiac cause was excluded based on normal electrocardiogram, 24-hour Holter monitoring and echocardiogram. Subsequently, on day three, CT angiogram of the circle of Willis was performed (Figure 1 [D-E]), which did not show an aneurysm or arteriovenous malformation. Further investigations done to exclude causes of secondary hypertension were non-contributory. His BP remained well controlled on Amlodipine 5 mg, throughout his period of hospitalization. As his workup did not reveal a convincing cause for his stroke beyond it occurring during sexual activity, a sleep study was performed which showed moderate sleep apnea with AHI of 15.

## Discussion

OSA is a common risk factor for hemorrhagic stroke which is often missed. Identifying OSA in a population is challenging when the common symptoms and signs such as excessive daytime sleepiness and snoring are often ignored by the patient [5, 6]. Given that patients with an unknown cause of stroke had an increased rate of SDB suggests that OSA may be a contributing factor to hemorrhagic stroke in this patient. Recently, Yoshida, et al. published a case of recurrent strokes secondary to OSA [7]. They used a “Trigger Sleep Blood Pressure monitoring (TSP) method”, which measures the BP of an individual when oxygen saturation falls below a set threshold. Using the TSP system they showed that there was a prominent surge in the patients nocturnal BP when his oxygen saturation dropped and resulted in the recurrence of hemorrhagic stroke [7]. Our patient had stroke at night and his BP was moderately elevated on arrival to hospital suggesting that he had a nocturnal BP surge because of OSA leading to hemorrhagic stroke even though he did not have any risk factors. Untreated OSA and high BP precipitated by sexual intercourse presumably led to increased intracranial pressure and bleeding in our patient [7].

Despite frequent compliance issues, Continuous Positive Airway Pressure (CPAP) is most commonly used as the standard treatment to lower the adverse outcomes of OSA after stroke [4]. However, our patient did not tolerate a trial of CPAP and was discharged home without it.

This case helps us to consider the alternative apnea modifying interventions and define the role of physicians in identifying and treating conditions like OSA, which could prevent serious complications as described above. All doctors should be aware of OSA as an independent risk factor for hemorrhagic stroke so that even in the patients with stroke, we can hope to prevent stroke recurrence by treating OSA [7].

## Conclusions

OSA may cause hemorrhagic stroke by a surge in nocturnal blood pressure during the periods of hypoxia. Consider taking a detailed sleep history for all patients with unexplained hemorrhagic stroke, especially if they have snoring, apneic episodes or other symptoms of OSA. If history is suggestive, it is necessary to do polysomnography in those patients at risk. Diagnosing and treating OSA could be critical in preventing hemorrhagic stroke and its recurrences.
